# On the Hole Injection for III-Nitride Based Deep Ultraviolet Light-Emitting Diodes

**DOI:** 10.3390/ma10101221

**Published:** 2017-10-24

**Authors:** Luping Li, Yonghui Zhang, Shu Xu, Wengang Bi, Zi-Hui Zhang, Hao-Chung Kuo

**Affiliations:** 1 Institute of Micro-Nano Photoelectron and Electromagnetic Technology Innovation, School of Electronics and Information Engineering, Hebei University of Technology, Key Laboratory of Electronic Materials and Devices of Tianjin, 5340 Xiping Road, Beichen District, Tianjin 300401, China; liluping1992hebut@hotmail.com (L.L.); zhangyh@hebut.edu.cn (Y.Z.); shu.xu@hebut.edu.cn (S.X.); wbi@hebut.edu.cn (W.B.); 2 Department of Photonics and Institute of Electro-Optical Engineering, National Chiao Tung University, Hsinchu 30010, Taiwan

**Keywords:** III-nitride semiconductor, multiple quantum well, light-emitting diode, hole injection efficiency, external quantum efficiency, internal quantum efficiency

## Abstract

The hole injection is one of the bottlenecks that strongly hinder the quantum efficiency and the optical power for deep ultraviolet light-emitting diodes (DUV LEDs) with the emission wavelength smaller than 360 nm. The hole injection efficiency for DUV LEDs is co-affected by the p-type ohmic contact, the p-type hole injection layer, the p-type electron blocking layer and the multiple quantum wells. In this report, we review a large diversity of advances that are currently adopted to increase the hole injection efficiency for DUV LEDs. Moreover, by disclosing the underlying device physics, the design strategies that we can follow have also been suggested to improve the hole injection for DUV LEDs.

## 1. Introduction

Compared to florescence-based deep ultraviolet emitters, III-nitride based deep ultraviolet light-emitting diodes (DUV LEDs) have shown a diversity of excellences including no mercury, low-voltage consumption, DC driving, small size and portability, and therefore they can be widely used in water sterilization, air purification, medical therapy, biological analysis et al. [1,2]. Currently, DUV LEDs emit the wavelength shorter than 360 nm, for which the external quantum efficiency (EQE) is still low (<10%) at the current stage. The EQE also continues to decrease as the AlN composition in the AlGaN quantum well further increases, and in other words, the optical performancefor DUV LEDs degrades as the emission wavelength is becoming shorter [3]. The poor EQE for DUV LEDs is ascribed to the low light extraction efficiency which is partly caused by the intrinsic and unique valence subband properties for the Al-rich AlGaN based quantum wells, i.e., the C–CH1 transition mainly produces TM-polarized DUV photons that propagate in parallel with the [0001]-faced quantum well plane [4,5,6,7]. The C–CH1 transition becomes more dominant as the emission wavelength further reduces. Another issue challenging the light extraction efficiency arises from the absorptive substrate and other passive layers with the energy band gap smaller than the emitted DUV photons [8,9,10]. The methods which are used to increase the light extraction efficiency have been reviewed and summarized by Park et al. [2]. Besides the light extraction efficiency, the EQE for DUV LEDs is also impacted by the internal quantum efficiency (IQE). According to the well-known ABC model [11], the IQE is partially determined by the defect-related non-recombination rate. The huge lattice and thermal mismatches between the sapphire substrate and the subsequent epi-layer cause a very large threading dislocation density (TDD) which is in the order of 10^9^–10^10^ cm^−2^ and causes the intrinsic internal quantum efficiency lower than 1% [1,3,12,13]. The Al-rich AlGaN based quantum wells for DUV LEDs possess no localized states and this makes the radiative emission very sensitive to the generated dislocations within the multiple quantum wells (MQWs) [1,14]. It is well known that the Auger recombination rate scales with the cubic power of the carrier density, and it is regarded as one of the origins causing the efficiency droop for III-nitride based blue and green LEDs [15]. However, as Piprek has summarized, the Auger recombination coefficient scales down as the energy band gap becomes large, e.g., in the case of Al-rich AlGaN quantum wells [16]. Hence, it is not clear if the Auger recombination plays an important role in influencing the IQE for DUV LEDs at the current stage [17]. The IQE is directly related to the generated photons from the MQW region. The generated photon number is decided by the radiative recombination rate, which is formulated by B × N^2^ (B denotes the radiative recombination coefficient and N represents the carrier concentration). The radiative recombination coefficient is remarkably influenced by the polarization induced electric field in the [0001] oriented AlGaN-based MQWs by means of spatially separating electron and hole wave functions [18,19,20]. The other concern comes from the injection efficiency for electrons and holes [11]. It is worth noting that both Si dopants and Mg dopants have the higher ionization energy for the Al-rich AlGaN epi-layer [21]. However, considering the even higher Mg ionization energy, the passivation effect by the hydrogen atoms during the metal organic chemical vapor deposition (MOCVD) growth and the even lower mobility [22,23], the hole injection and the electron injection are not synchronized. Note, the electron concentration for the Al-rich n-AlGaN layer can still reach the level of ~10^18^ cm^−3^, and therefore a satisfied electron concentration in the MQW can still be acquired [24]. Moreover, the TDD has also been substantially reduced to ~10^8^ cm^−2^ by improving the crystal growth technology [25,26,27,28,29,30,31,32], which enables a high intrinsic internal quantum efficiency of 50~80% that is obtained by conducting the low-temperature photoluminescence measurement [3,13]. As a result, a systematic review on the proposals to increase the hole injection capability for DUV LEDs is essentially necessary at this time. It is also worthy of noting that an enhanced hole concentration in the MQW region enables a screened polarization induced electric field therein [33].

**Figure 1:**
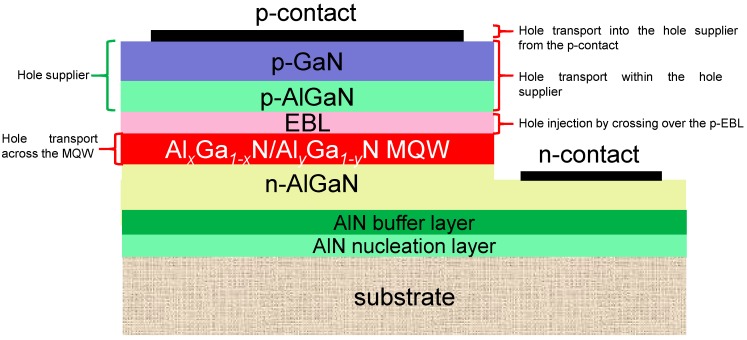
Schematic device architecture for a typical AlGaN based DUV LED and the four key factors that co-decide the hole injection efficiency. The hole supplier contains the p-AlGaN layer and the p-GaN layer.

The schematic structure for a typical AlGaN based DUV LED that is grown on the sapphire substrate is shown in Figure 1. A thin AlN nucleation layer is firstly grown on the sapphire substrate, and then an AlN buffer layer is subsequently grown to form a two-dimensional (2D) film that serves as the growth template for the following epi-layers. Next, a Si-doped n-AlGaN layer is grown on the AlN template to provide electrons. The DUV photons are generated by the Al*_x_*Ga_1−*x*_N/Al*_y_*Ga_1−*y*_N (*x* < *y*) MQWs which are grown on the n-AlGaN layer. To prevent the electron escape from the MQW region, a p-AlGaN electron blocking layer (EBL) is then grown on the MQW. The AlN composition for the p-EBL shall be higher than that for the AlGaN quantum barrier. The p-EBL is afterwards capped with a p-AlGaN/p-GaN heterojunction, which serves as the hole supplier and the p-GaN layer enables the formation of the p-type ohmic contact at the same time. The AlN composition of the p-AlGaN region for the hole injection layer is smaller than that of the p-EBL.

Figure 1 shows that the holes firstly are injected into the hole supplier from the p-type ohmic contact. Then the holes travel across the p-EBL before entering the MQW region. We then will make discussions uncovering the important factors that limit the hole injection. To guarantee a smooth hole injection, one shall (1) increase the hole tunneling efficiency from the p-type ohmic contact into the hole supplier; (2) improve the hole transport within the hole supplier; (3) reduce the hole blocking effect that is caused by the p-EBL; (4) increase the hole concentration in the MQWs. In this work, we also will conduct in-depth review on the various proposed approaches to increase the hole injection capability for DUV LEDs.

## 2. Increase the Hole Injection Efficiency from the p-Type Ohmic Contact into the Hole Supplier

The first obstacle that the injected holes have to overcome arises from the p-GaN/p-ohmic contact interface [34]. One approach to increase the hole injection at the p-GaN/p-ohmic contact interface is to engineer the p-type metals and improve the intraband tunneling efficiency at the interface. Normally Ni metal is deposited on the surface of the hole injection layer and then it is annealed in the ambient of O_2_ to form the p-type NiO*_x_* material, which serves as the interfacial layer and is very helpful to enable the ohmic contact (i.e., the current-voltage curve lacks the rectifying property) [35,36,37] while the additional Au metal on the p-type NiO*_x_* layer improves the conductivity of the ohmic contact [38]. The Ni/Au p-type contact has been widely adopted due to the mature deposition and annealing processes. Chae et al. propose Zn/Ni alloy to further improve the ohmic property and the transparency [39], since the Zn/Ni alloyed metal serves as the hydrogen collector and can well absorb the hydrogen that is detached from the Mg–H bonds during the annealing process. Kalaitzaki et al. also try Cr and Pd to obtain the p-type ohmic contact [40]. Jang et al. show the effectiveness of Ru, Ir, Ru/Ir alloy Ir/Ni alloy in improving the p-type ohmic feature [41]. By properly optimizing the annealing conditions, the outdiffusion of Ga atoms from the p-GaN surface results in a decent number of Ga vacancies. Ga vacancies can well trap the electrons, and thus the holes are less compensated by the electrons, resulting in the accumulated hole concentration at the p-GaN surface [42]. The high hole concentration at the p-GaN surface can remarkably reduce the intraband tunneling region width and hence improve the hole tunneling efficiency. 

**Figure 2:**
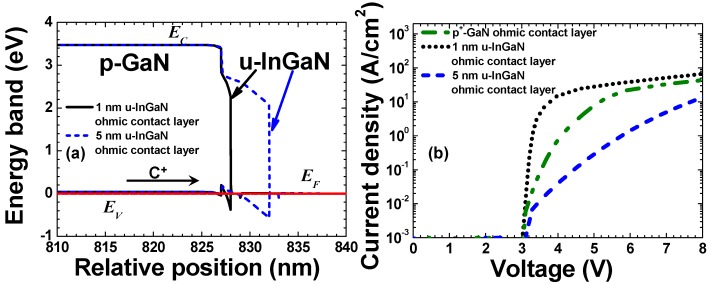
(**a**) Energy band diagrams at the equilibrium state for u-InGaN/p-GaN structures with the u-InGaN layer thicknesses of 1 nm and 5 nm, respectively; (**b**) calculated current in terms of the applied voltage for LEDs with p^+^-GaN, 1 nm thick u-InGaN and 5 nm thick u-InGaN as the ohmic contact layers, respectively. Here, u-InGaN, *E_C_*, *E_V_* ad *E_F_* represent the undoped InGaN cap layer, the conduction band, the valence band and the Fermi level, respectively.

From the point view of the device design, the hole injection efficiency at the p-GaN/p-ohmic contact interface can be improved by properly managing the polarization effect. On one hand, the increase of the Mg doping efficiency can be realized by adopting a short-period superlattice structure [43,44]. The ability for the holes to transport from the p-type ohmic contact into the hole injection layer can be promoted if the superlattice structure is utilized as the ohmic contact layer, e.g., p-InGaN/GaN superlattice [45], p-GaN/AlN superlattice [46]. However, a p-type material with a lower energy bandgap has to be capped on the superlattice structure that has a large energy bandgap, e.g., p-Al*_x_*Ga_1−*x*_N/Al*_y_*Ga_1−*y*_N (*x* < *y*) superlattice with the thin p-Al*_x_*Ga_1−*x*_N as the cap layer [47,48,49], p-AlN/AlInGaN supperlattice with the thin p-AlInGaN as the cap layer [50,51,52], which structures are also able to improve the hole transport within the hole supplier and the relevant discussions will be conducted subsequently. On the other hand, the enhanced hole injection from the p-type ohmic contact into the hole supplier for LEDs can be realized by using a polarized cap layer [53,54,55]. If we take the [0001] oriented InGaN/p-GaN structure for example (see Figure 2a), the origin for the polarized cap layer in improving hole injection efficiency lies on the fact that the polarization effect will bend the energy band of the InGaN layer in the way favoring the intraband tunneling process for the carriers. When compared with the p^+^-GaN as the ohmic contact layer, such energy band bending in the thin InGaN cap layer is decided by the polarization induced electric field. Thus the surface depletion region width is precisely determined by the InGaN cap layer thickness. If we make the InGaN cap layer thin (e.g., 1 nm in this case), the hole tunneling efficiency can be significantly enhanced, leading to the improved electrical conductivity for the LED with the proposed design (see Figure 2b). Figure 2a also illustrates that if the InGaN cap layer is further thickened, the surface depletion region width will become larger and the intraband tunneling efficiency for the carriers becomes low. As a result, the calculated current-voltage characteristic in Figure 2b shows an even more degraded electrical conductivity for the LED with the 5 nm thick InGaN cap layer than that for the LED with p^+^-GaN as the ohmic contact layer. The proposed design can be further evolved into InGaN/p-AlGaN structure which proves to be effective in improving the hole injection and enhancing the optical power for AlGaN based UV LEDs [55].

**Figure 3:**
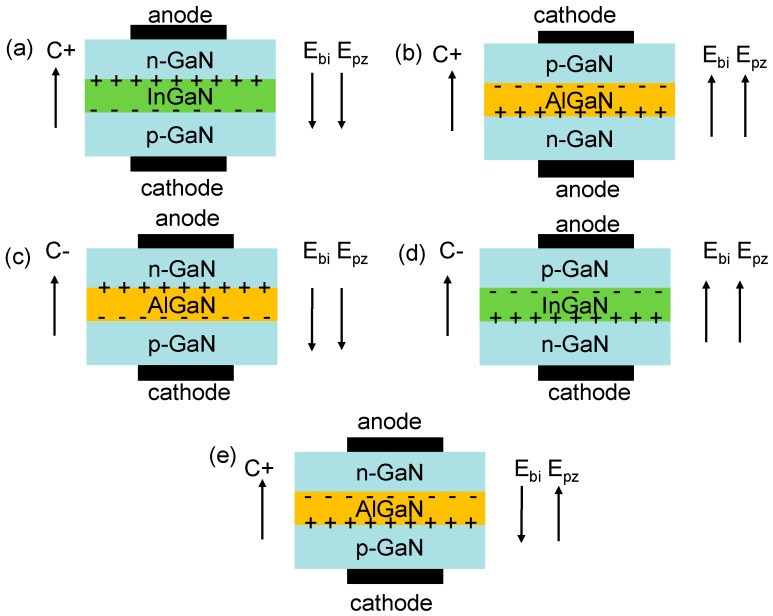
(**a**) [0001] oriented n-GaN/InGaN/p-GaN polarization tunnel junction; (**b**) [0001] oriented p-GaN/AlGaN/n-GaN polarization tunnel junction; (**c**) [000-1] oriented n-GaN/AlGaN/p-GaN polarization tunnel junction; (**d**) [000-1] oriented p-GaN/InGaN/n-GaN polarization tunnel junction and (**e**) [0001] oriented dielectric-constant-controlled (DCC) tunnel junction. E_bi_, E_pz_ represent the built-in electric field and the polarization induced electric field, respectively.

**Figure 4:**
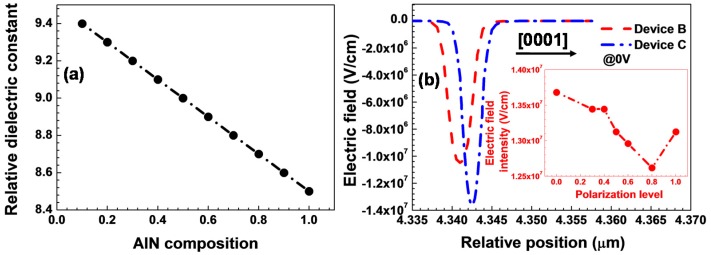
(**a**) Relative dielectric constant of the AlGaN layer in terms of the AlN composition; (**b**) electric field profiles within the tunnel regions for the UV LED (i.e., Device B) with the conventional n^+^-GaN/p^+^-GaN tunnel junction and the UV LED (i.e., Device C ) with the DCC tunnel junction. The positive direction for the electric field is along the [0001] direction. The inset shows the electric field intensity as a function of the polarization level. Reproduced from Ref. [67], with the permission of Wiley.

The other proposal to enable the efficient hole injection from the p-type ohmic contact into the hole supplier is to utilize a tunnel junction [56,57,58]. The advantage of the tunnel junction is a direct contact between the n^+^-GaN layer and the metal, since the n^+^-GaN layer has a much higher doping efficiency than the p^+^-GaN layer. Therefore, by means of the tunnel junction, the hole injection efficiency from the external metal contact into the device can be improved, and this can result in the enhanced quantum efficiency. However, the shortcoming for the LED with the conventional tunnel junction lies on the larger forward voltage drop which is due to the poor Mg doping efficiency for the p^+^-GaN layer. Fortunately, the forward voltage drop across the tunnel junction can be decreased by using a polarization tunnel junction [59,60,61]. The polarization induced electric field within the polarization tunnel junction has to be along the same direction as the built-in electric field that is generated by the ionized dopants in the n-type and p-type layers. Here it is worthy of defining the structures of the polarization tunnel junctions (see Figure 3a–d). If the growth direction is along the [0001] orientation, then the suggested structures of the polarization tunnel junction are shown in Figure 3a,b. The anode and the cathode have to be placed on the top n-GaN layer and the bottom p-GaN layer, respectively for Figure 3a, which structure has been extensively discussed in Refs. [59,60] and is utilized in blue and UV III-nitride LEDs [62,63,64,65,66]. However, if the polarization tunnel region is AlGaN-based (see Figure 3b), then the structure stack has to be anode/n-GaN/AlGaN/p-GaN/cathode to ensure that both the built-in electric field and the polarization induced electric field are along the same direction. However the application of such AlGaN based polarization tunnel junction in III-nitride based LEDs is rare partly due to the difficulty in growing the MQWs and the n-type electron source layers on the p-type layer. Figure 3c,d show the configurations of the polarization tunnel junction grown along the [000-1] orientation. The design strategies are the same as those in Figure 3a,b, i.e., to guarantee that the polarization induced electric field is along the same direction as the built-in electric field. Hence details will not be addressed here. Note, investigations on the [000-1] oriented polarization tunnel junctions and the applications in III-nitride LEDs are rarely conducted at the current stage. However, we have reported a dielectric-constant-controlled (DCC) tunnel junction for UV LEDs, and the structure of the DCC tunnel junction is presented in Figure 3e [67]. It is worthy of noting that the polarization induced electric field within the DCC tunnel junction opposes that of the built-in electric field, i.e., the ionized dopants will be partially compensated by the polarization induced interface charges. Fortunately, the net electric field intensity is also partially decided by the relative dielectric constant (*ε_r_*) of the tunnel junction, and the *ε* decreases as the AlN composition for the AlGaN tunnel layer increases (see Figure 4a). A decreased *ε_r_* can increase the electric field intensity and favors the interband tunneling. In our case, the polarization level is set to 40% and the AlN composition used in the tunnel junction is 30%. According to Figure 4b, when compared with the conventional n^+^-GaN/p^+^-GaN tunnel junction, the DCC tunnel junction possesses the increased electric field intensity. Hence, the optical power and the internal quantum efficiency for the UV LED with the DCC tunnel junction have been improved. Note, the inset of Figure 4a also shows the electric field intensity within the tunnel region in terms of different polarization levels, from which we can see that the electric field intensity decreases as the polarization level increases till it reaches 80%. The decrease of the electric field intensity is ascribed to the compensation effect to the ionized dopants by the polarization induced interface charges (see Figure 3e). However, if the polarization level further increases to 100%, more ionized dopants will be compensated by the polarization induced charges which will simultaneously increase the depletion region width in the n^+^-GaN and p^+^-GaN layers. Thus more dopants will be ionized and the electric field intensity becomes high. Nevertheless, the enhanced electric field intensity when the polarization level is 100% sacrifices the tunnel region width and hence is not advisable for achieving the high-efficiency hole injection. The inset figure in Figure 4b also illustrates that, besides the *ε_r_* for the tunnel region, the polarization induced charge density within the DCC tunnel junction is essentially important, and this requires the precise control for the AlN composition and the crystal relaxation.

## 3. Enhance the Hole Transport within the Hole Supplier

Another bottleneck hindering the hole injection efficiency originates from the hole supplier. According to Figure 1, the hole supplier for the DUV LED comprises of the p-AlGaN/p-GaN heterojunction. The valence band discontinuity at the p-AlGaN/p-GaN heterojunction hinders the hole injection. Kuo et al. suggest the stair-cased (p-AlGaN)*_n_*/p-GaN heterojunction to facilitate the hole transport [68]. Here, the AlN composition for the stair-cased (p-AlGaN)*_n_*/p-GaN heterojunction decreases along the [0001] growth orientation and *n* represents the total number of the stair-cased p-AlGaN layers. As has been demonstrated previously, the Mg doping efficiency for the Al-rich p-AlGaN layer is even lower and that will lead to a low hole concentration for the hole supplier. However, the suggested structure by Kuo et al. seems of less contribution to increasing the hole concentration for the hole supplier. A promising approach to increase the hole concentration is to utilize a superlattice structure, such as the p-Al*_x_*Ga_1−*x*_N/Al*_y_*Ga_1−*y*_N superlattice [47,48,49], the p-AlN/AlInGaN superlattice [50,51,52], the polarization induced electric field of which will become high and enables an enhanced Mg ionization efficiency. According to the report by Cheng et al. [48], the activation energy for Mg dopants is as low as 17 meV for the p-Al*_x_*Ga_1−*x*_N/Al*_y_*Ga_1−*y*_N (*x* > *y*) superlattice with the average AlN composition of 60% when compared with the activation energy of 146 meV and 323 meV for the p-GaN layer and the p-Al_0.70_Ga_0.30_N layer, respectively. More importantly the hole concentration for the p-type superlattice structure is less affected by the temperature. The observation also agrees well with the reports by Simon et al. [69] and Zhang et al. [70], respectively. Both of the two groups use the polarization induced electric field within the p-AlGaN layer with a graded AlN composition to increase the Mg doping efficiency and form the three dimensional hole gas (3DHG). Most recently, indium-surfactant-assisted Mg-delta doping has been reported to enhance the Mg-doping efficiency in the p-AlGaN layer by Chen et al. [71]. The measured hole concentration is as high as 4.75 × 10^18^ cm^−3^ by using the proposed technique. The underlying mechanism for the enhanced hole concentration is the coupled effect of the high Mg incorporation efficiency, the less compensation effect and the indium-caused energy band modulation effect. The less compensation effect is owing to the indium surfactant because the indium desorption leaves the vacancies for Mg dopants to occupy. Moreover, the incorporation of the partial indium into the crystal modulates the energy band and enables the polarization-induced electric field to form the two-dimensional hole gas (2DHG).

**Figure 5:**
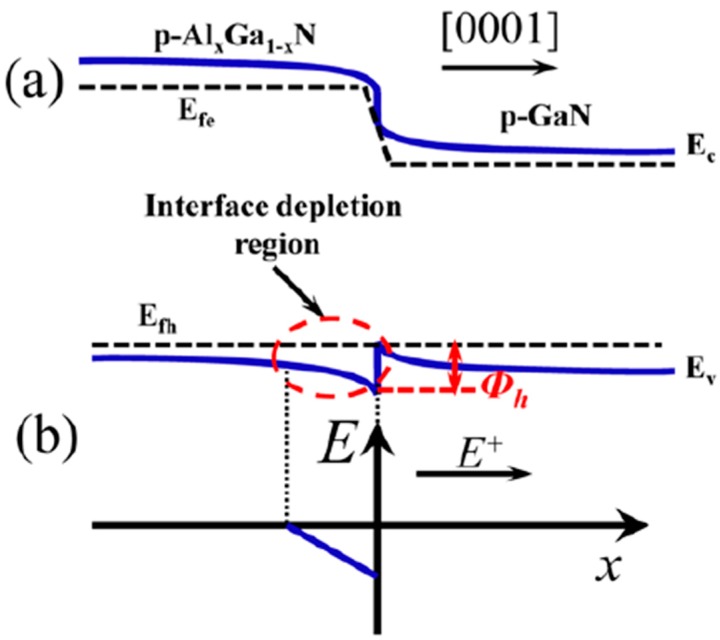
(**a**) Schematic energy band diagram for the electric field reservoir that consists of the p-Al*_x_*Ga_1−*x*_N/p-GaN heterojunction, which possesses the interface depletion region; (**b**) schematic electric field profile within the electric field reservoir. The positive direction of the electric field is along the [0001] orientation. E_c_, E_v_, *Ø_h_*, E_fe_ and E_fh_ denote the conduction band, the valence band, the valence band barrier height caused by the p-Al*_x_*Ga_1−*x*_N layer, quasi-Fermi levels for electrons and hole, respectively. Reproduced from Ref. [72], with the permission of Optical Society of America.

The other method to increase the hole transport within the hole supplier is to increase the drift velocity and the kinetic energy for holes. Previously, we have proposed and discussed an electric field reservoir [72]. The electric field reservoir is depicted in Figure 5. The electric field reservoir has to possess an interface depletion region which can trigger the electric field in the depletion region. Such electric field will not be screened by the free carriers, and hence the holes are able to continuously receive the energy from the electric field. However, if the AlN composition for the p-Al*_x_*Ga_1−*x*_N layer is too high, then the aforementioned interface depletion region will further extend the width into the p-Al*_x_*Ga_1−*x*_N layer, and this will significantly deplete the holes and causes a low hole concentration in the p-Al*_x_*Ga_1−*x*_N layer. Hence, to better understand the impact of the electric field reservoir on the hole injection, parametric investigations have to be conducted.

**Figure 6:**
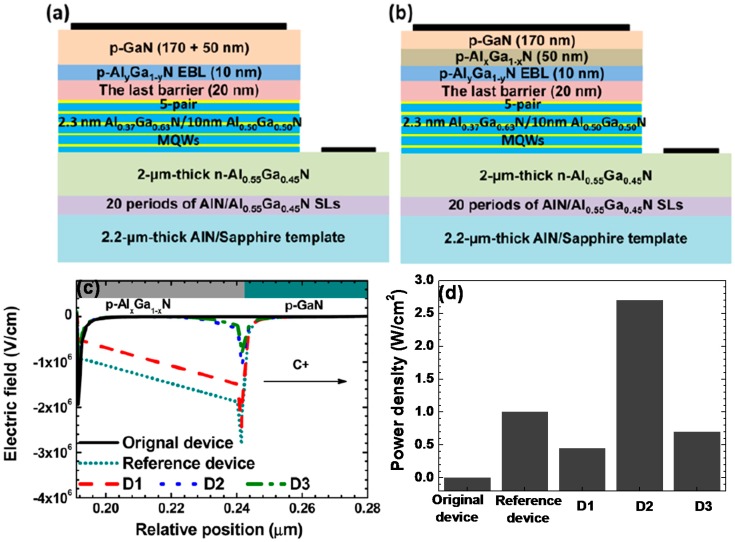
(**a**) Schematic DUV LED structure for the Original device that has no p-Al*_x_*Ga_1−*x*_N layer; (**b**) schematic DUV LED structures for the Reference device, Device 1 (D1), Device 2 (D2) and Device 3 (D3); (**c**) numerically calculated electric field profiles in the hole suppliers for the studied DUV LEDs, and (**d**) summarized optical output power for the five investigated DUV LEDs. Detailed AlN compositions of the p-EBLs and the p-Al*_x_*Ga_1−*x*_N layers for the studied devices are shown in Table 1. Figure 6a–c are reproduced from Ref. [72], with the permission of Optical Society of America. Figure 6d is summarized according to the reported values in Ref. [72].

Figure 6a,b present the studied DUV LEDs with different device architectures. The studied devices differ among each other in the p-EBL and the hole supplier, and detailed structure information can be found in Table 1. Clearly we can see from Table 1 that the *Ø_h_* decreases as the AlN composition in the p-Al*_x_*Ga_1−*x*_N layer reduces. Meanwhile, the electric field profiles within the hole suppliers for the tested DUV LEDs are presented in Figure 6c, from which we can see that the electric field is small in the p-GaN layer for the Original device. Nevertheless, if the hole supplier consists of the p-Al*_x_*Ga_1−*x*_N/p-GaN heterojunction, the strong electric field is produced in the interface depletion region, which can well increase the drift velocity and the corresponding kinetic energy for the incoming holes. By following $W=e\times \int_{0}^{l}E_{field}\times dx$, the work (*W*) that the holes obtain can be calculated (see Table 1). Here *e*, *l* and *E_field_* represent the unit electronic charge, the integration range and the electric field within the integration range [73,74]. Figure 6c also shows that the p-Al*_x_*Ga_1−*x*_N layer is fully depleted for the Reference device and Device 1 (D1), and therefore the electric field intensity is very strong which produces an extremely large energy for holes (see Table 1). If the AlN composition decreases, then the interface depletion region shrinks and this simultaneously makes the holes receive less energy from the electric field reservoir. Fortunately, the energy that the holes obtain is still high when compared with the holes for the Original device. Meanwhile, since the interface depletion region in the p-Al*_x_*Ga_1−*x*_N layer is small (e.g., Device 2 (D2)), a high hole concentration therein can still be maintained. Because of the above two reasons, the optical power for Device (D2) is the strongest. To summarize, to maximize the function of the electric-field reservoir in favoring the hole injection, one shall make holes obtain sufficient energy while keeping a decent hole concentration in the hole supplier.

## 4. Reduce the Hole Blocking Effect by the p-EBL

The holes arrive at the p-EBL after completing the trip in the hole supplier. The valence band discontinuity between the p-EBL and the hole supplier can strongly block the hole injection. Considering the relevance between the carrier concentration and the energy band barrier height [75], one promising approach to reduce the hole blocking effect caused by the p-EBL is to increase the Mg doping efficiency for the hole supplier. As mentioned in the previous discussions, the Mg doping efficiency in the hole supplier can be enhanced by using superlattice structures [47,48,49,50,51,52], 3DHG structure [69,70,76] and the indium-surfactant-assisted Mg-delta doping technique [71]. 

**Figure 7:**
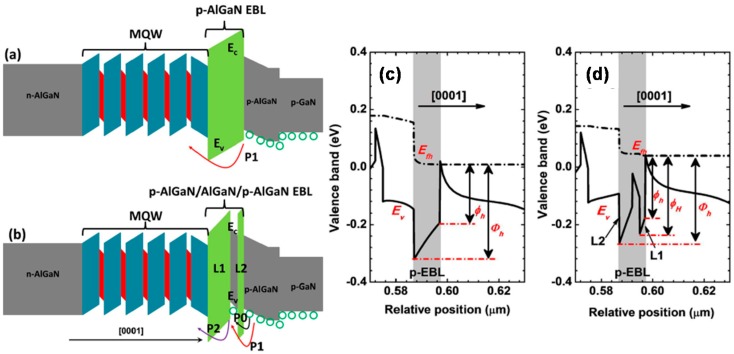
Schematic energy band diagrams for (**a**) Device A with the conventional bulk p-EBL and (**b**) Device B with the proposed p-EBL. Numerically calculated valence bands in the vicinity of (**c**) the conventional p-EBL for Device A and (**d**) the proposed p-EBL for Device B. In Figure 7a, P1 represents the hole transport by the thermionic emission. In Figure 7b, P0 denotes the hole transport by the intraband tunneling process, P1 and P2 mean the hole thermionic emission from the hole supplier into the thin AlGaN insertion layer and from the AlGaN insertion layer into the MQW region, respectively. Reproduced from Ref. [24], with the permission of American Chemical Society.

It is unambiguous that the hole injection across the p-EBL can be improved once the valence band barrier height for the p-EBL decreases. For that purpose, it is advisable to relocate the superlattice structure to the p-EBL region to form the superlattice p-EBL [77,78]. Another strategy to increase the hole injection is to facilitate the hole tunneling process, which can be realized by reducing the thickness for the p-EBL while increasing the AlN composition for the p-EBL, e.g., using the AlN based p-EBL with a properly small thickness [79]. To acquire the intraband-tunneling-assisted hole injection, we have recently proposed a p-EBL with a very thin insertion layer that has a smaller energy band gap for the DUV LED [24], and the studied device structures are shown in Figure 7a,b, respectively. It is worth noting that the proposed p-EBL structure is easier to achieve experimentally. If the DUV LED possesses the bulk p-EBL, the major transport for holes is via the thermionic emission, which is strongly affected by the valence band offset and the hole concentration, i.e., *Φ_h_* = *ΔE_V_* − *kT* × *ln*(*p*/*N_V_*) [80]. Here, *Φ_h_*, *ΔE_V_*, *k*, *T*, *p* and *N_V_* represent the valence band barrier height, the valence band offset, the Boltzmann constant, the carrier temperature, the hole concentration and the effective density of states for holes, respectively. Therefore, it is suggested to reduce *Φ_h_* by increasing the hole concentration (*p*). Fortunately, the hole concentration can be increased if a very thin AlGaN layer with a smaller energy bandgap is inserted into the p-EBL. More importantly, the thin AlGaN insertion layer has to be close to the hole supplier, i.e., layer L2 in Figure 7b has to be made thin. Once layer L2 is thin, the holes can be injected into the AlGaN insertion layer by both the thermionic emission and the intraband tunneling process. As a result, a high local hole concentration can be readily obtained in the thin AlGaN insertion layer, which helps to decrease the valence band barrier height for layer L1 (see Figure 7b). The valence bands of the p-EBLs for the two investigated DUV LEDs are shown in Figure 7c,d, respectively. The values for the valence band barrier heights are summarized and demonstrated in Table 2, from which we can clearly see that the valence band barrier height that is symbolized by *Φ_h_* for the proposed p-EBL turns smaller. It is worth noting that the larger *Ø_h_* for the proposed p-EBL reflects a lower hole accumulation at the p-EBL/p-AlGaN interface according to the aforementioned equation. The lower hole accumulation at the p-EBL/p-AlGaN interface for the proposed DUV LED is well ascribed to the excellent hole tunneling effect into the thin AlGaN insertion layer for the proposed p-EBL. 

**Figure 8:**
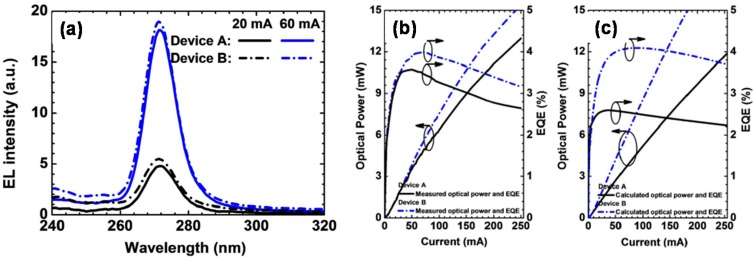
(**a**) Experimentally measured EL spectra; (**b**) experimentally measured EQE and optical power, and (**c**) numerically calculated EQE and optical power for Devices A and B, respectively. Reproduced from Ref. [24], with the permission of American Chemical Society.

Because of the reduced blocking effect for the suggested p-EBL and the more favored hole injection simultaneously, the hole concentration in the active region increases and this translates to the enhanced radiative recombination. As a result, the measured intensity for the electroluminescence (EL) spectra for Device B with the proposed p-EBL is higher than that for Device A with the conventional bulk p-EBL (see Figure 8a). Figure 8b,c demonstrate the measured and calculated optical power and EQE for Devices A and B, respectively. Both experimentally and numerically, the optical power and the EQE are improved for Device B thanks to the improved hole injection efficiency. Meanwhile, the consistence between the measured and calculated results validates the adopted physical models when conducting the numerical computations. 

## 5. Increase the Hole Concentration in the MQW Region

For In*_x_*Ga_1−*x*_N/GaN based visible LEDs, it has been widely admitted that the holes are strongly accumulated in the quantum well close to the p-EBL side [11]. However, the hole distribution across the active region shows different spatial profiles for III-nitride based DUV LEDs according to reports by different groups [24,68,72,81,82,83,84], e.g., the highest hole concentration is not always found in the quantum well closest to the p-EBL [68,82]. Tsai et al. attribute the interesting observations to the reduced valence band offset for the Al*_x_*Ga_1−*x*_N/Al*_y_*Ga_1−*y*_N (*x* < *y*) based quantum wells, which therefore is beneficial for the hole transport across the MQW region [82], i.e., the valence band offset is 0.185 eV for the Al_0.55_Ga_0.45_N/Al_0.72_Ga_0.28_N quantum wells of Tsai et al. and 0.210 eV for the In_0.15_Ga_0.85_N/GaN quantum wells with the ~450 nm emission wavelength [11], respectively. Till now, various conduction band offset/valence band offset ratios (*ΔE_C_*/*ΔE_V_*) are assumed when calculating the carrier transport within the Al*_x_*Ga_1−*x*_N/Al*_y_*Ga_1−*y*_N (*x* < *y*) based quantum wells, e.g., 70/30 [81,83,84,85], 65/35 [68,82] and 50/50 [24,72]. We agree to the point proposed by Tsai et al., since we find that the hole concentration in quantum well closest to the p-EBL becomes high if the *ΔE_C_/ΔE_V_* of 50/50 is adopted. Although a larger valence band offset (i.e., *ΔE_C_/ΔE_V_* = 50/50) is suggested for AlGaN/AlGaN interface [16], it seems that the hole distribution in the Al*_x_*Ga_1−*x*_N/Al*_y_*Ga_1−*y*_N (*x* < *y*) based quantum wells is much more uniform than that in the In*_x_*Ga_1−*x*_N/GaN based quantum wells. In addition, the quantum barrier for DUV LEDs is also AlGaN based, for which the AlN composition can be optimized and this will provide more freedom in further modifying the hole distribution within the active region [84]. 

Judging from the previously published reports so far, it is seemingly more realistic to increase the hole concentration in each Al*_x_*Ga_1−*x*_N/Al*_y_*Ga_1−*y*_N (*x* < *y*) based quantum well rather than homogenize the hole distribution across the Al*_x_*Ga_1−*x*_N/Al*_y_*Ga_1−*y*_N (*x* < *y*) based quantum wells. To achieve that goal, staggered quantum wells have never been proposed and numerically shown the effectiveness in increasing the hole concentration in the Al*_x_*Ga_1−*x*_N/Al*_y_*Ga_1−*y*_N (*x* < *y*) based quantum wells [81,83]. Another concern affecting the hole concentration is the thickness for the quantum well and the quantum barrier. The recommended quantum well thickness is smaller than 2 nm for DUV LEDs [86,87,88,89], the quantum well of which thickness can keep both the high hole concentration and the excellent spatial overlap for electron-hole wave functions [86]. Though specific discussions regarding the quantum barrier thickness on the hole concentration in the quantum wells are not conducted yet, Refs. [86,87,88,89] employ the quantum barriers with the thickness smaller than 8 nm, and this is quite different from the InGaN/GaN based visible LEDs, which normally utilize the barrier thickness lager than 10 nm [11]. Kim et al. then numerically propose that a thinner p-side quantum barrier thickness and a thicker n-side quantum barrier can further increase the hole concentration level in the quantum wells [85]. The findings by Kim et al. are agreeable with the report by Tsai et al. [82]. In spite of the high hole concentration in the quantum wells for the DUV LED with the thinner p-side barrier and the thicker n-side barrier, the DUV LED does not produce the strongest internal quantum efficiency when compared to the device with the thicker p-side barrier and the thinner n-side barrier. This is due to fact that the DUV LED with the thinner p-side barrier and the thicker n-side barrier does not possess the high electron confinement efficiency. 

It is worth noting that, thus far, there are hardly any reports fully concentrating on the influences of the *ΔE_C_/ΔE_V_*, the combination of the AlN composition for the quantum well and the quantum barrier, the combination of the thickness for the quantum well and the quantum barrier, and the quantum well number etc. on the hole transport within the active region for DUV LEDs with various emission wavelengths (e.g., 250~300 nm that is widely used in water and air sterilization). Therefore, there is still much space left for the DUV LED community to even better understand the physics for the carrier transport within the active region and to further optimize the MQWs before getting the satisfied hole concentration for DUV LEDs.

## 6. Summary

This work has comprehensively reviewed the current techniques to increase the hole injection efficiency and improve the quantum efficiency for AlGaN based DUV LEDs. The hole concentration in the MQW region is co-determined by (1) the p-type ohmic contact/the hole supplier interface; (2) the hole supplier; (3) the p-EBL and (4) the active region. 

Besides engineering the p-metals, the barrier that hinders the hole injection at the p-type ohmic contact/the hole supplier interface can be solved by using the polarization effect for [0001] oriented heterojunction, e.g., the polarized cap layer, the polarization tunnel junction and the dielectric-constant-controlled tunnel junction. With the aid of the existing proposed technologies, it is also worth investigating the possibility of realizing the p-type ohmic contact without involving the p-GaN cap layer in the hole supplier for DUV LEDs, and by doing so the light extraction efficiency and the external quantum efficiency can be further improved. The hole transport within the hole supplier can be more favored if the hole concentration in the hole supplier increases by adopting the superlattice doping approach. Moreover, we also propose the electric field reservoir to accelerate the holes and the relevant device physics has also been thoroughly demonstrated. Then it is necessary to find the alternatively designed hole supplier in which the concentration and the kinetic energy for holes can be both kept high. The hole blocking effect inducedby the p-EBL can be reduced either/both by increasing the Mg doping efficiency or/and managing the hole transport by enhancing the intraband tunneling efficiency. To meet the goal of the high Mg doping efficiency and the promoted hole tunneling capability, the superlattice p-EBL at the current stage seems to be an option as long as that structure can be very well managed. Last but not the least, according to the reviewed works, we realize that the holes are more homogenized in the AlGaN based quantum wells for DUV LEDs when compared with the InGaN based quantum wells for visible LEDs, such that a significant hole accumulation in the quantum well closest to the p-EBL is not observed for DUV LEDs. Therefore, the target is to enhance rather than homogenize the hole concentration in the MQW region for DUV LEDs. However, according to our point of view, a comprehensive study regarding the impact of different types of MQWs on the hole concentration levels for DUV LEDs with different wavelengths lacks at the moment. Therefore, more efforts shall be paid to further reveal how the different designs of the active region affect the hole concentration for DUV LEDs, and the in-depth device physics shall also be uncovered and summarized.

Considering various epi-growth technologies by different groups, uncertain physical parameters (e.g., band offset ratio for Al*_x_*Gal_1−*x*_N/Al*_y_*Ga_1−*y*_N (*x* < *y*) based quantum wells) and different mathematical mesh settings by different simulation people, it is less possible to conclude the best design at this momentby means of comparing and summarizing the proposals reported by different groups. Therefore, this requires a systematic and consistent study to reveal the sensitivity of the hole injection on different DUV structures, and we suggest numerical calculations, since the consistence of numerical DUV LED structures is easier to control than that for experimentally grown DUV LEDs.

## Figures and Tables

**Table 1:** AlN compositions of the p-EBLs and the p-Al*_x_*Ga_1−*x*_N layers, values of the *Ø_h_* and the work (*W*) that is conducted on the holes by the respective electric field reservoir for all the studied DUV LEDs. Reproduced from Ref. [72], with the permission of Optical Society of America.

Devices	p-Al*_x_*Ga_1−*x*_N	*Φ_h_* (meV)	p-Al*_y_*Ga_1−*y*_N	Work (meV)
Original device	p-GaN (50 nm)	0	p-Al_0.68_Ga_0.32_N EBL (10 nm)	−277.50
Reference device	p-Al_0.49_Ga_0.51_N (50 nm)	583.00	p-Al_0.68_Ga_0.32_N EBL (10 nm)	−7454.70
Device 1 (D1)	p-Al_0.49_Ga_0.51_N (50 nm)	460.00	p-Al_0.60_Ga_0.40_N EBL (10 nm)	−5456.10
Device 2 (D2)	p-Al_0.40_Ga_0.60_N (50 nm)	322.00	p-Al_0.68_Ga_0.32_N EBL (10 nm)	−381.97
Device 3 (D3)	p-Al_0.30_Ga_0.70_N (50 nm)	238.00	p-Al_0.68_Ga_0.32_N EBL (10 nm)	−365.72

**Table 2:** Values of different valence band barrier heights for the two p-EBLs. Reproduced from Ref. [24], with the permission of American Chemical Society.

-	Device A	Device B
*ϕ_h_* (meV)	~206.51	~217.40
*ϕ_H_* (meV)	-	~234.64
*Φ_h_* (meV)	~335.18	~303.41
